# Hydroclimatic changes of Lake Bosten in Northwest China during the last decades

**DOI:** 10.1038/s41598-018-27466-2

**Published:** 2018-06-14

**Authors:** Junqiang Yao, Yaning Chen, Yong Zhao, Xiaojing Yu

**Affiliations:** 1 Institute of Desert Meteorology, Desert Meteorology Field Scientific Experimental Bases of The Taklimakan Desert, China Meteorological Administration, Urumqi, 830002 China; 2 State Key Laboratory of Desert and Oasis Ecology, Xinjiang Institute of Ecology and Geography, Chinese Academy of Sciences, Urumqi, 830011 China; 3 School of Atmospheric Science, Chengdu University of Information Technology, Chengdu, 610225 China

## Abstract

Bosten Lake, the largest inland freshwater lake in China, has experienced drastic change over the past five decades. Based on the lake water balance model and climate elasticity method, we identify annual changes in the lake’s water components during 1961–2016 and investigate its water balance. We find a complex pattern in the lake’s water: a decrease (1961–1987), a rapid increase (1988–2002), a drastic decrease (2003–2012), and a recent drastic increase (2013–2016). We also estimated the lake’s water balance, finding that the drastic changes are caused by a climate-driven regime shift coupled with human disturbance. The changes in the lake accelerated after 1987, which may have been driven by regional climate wetting. During 2003 to 2012, implementation of the ecological water conveyance project (EWCP) significantly increased the lake’s outflow, while a decreased precipitation led to an increased drought frequency. The glacier retreating trend accelerated by warming, and caused large variations in the observed lake’s changes in recent years. Furthermore, wastewater emissions may give rise to water degradation, human activity is completely changing the natural water cycle system in the Bosten Lake. Indeed, the future of Bosten Lake is largely dependent on mankind.

## Introduction

As an important component of the hydrological cycle, lakes influence many aspects of the environment, including its ecology, biodiversity, economy, wildlife and human welfare^1–3^. Lakes remain highly sensitive to climate change and human activities, so it can serve as an important proxy of regional climate change and anthropogenic impact^4–7^. Freshwater lakes in arid regions play a special role, not only because of their valuable, yet limited, water resources, but they also have regional ecological and environmental functions^8^. For example, alpine lakes provide a link between the hydrosphere, atmosphere, cryosphere, biosphere, and anthroposphere.

The Tianshan Mountains are regarded as a main water tower in Central Asia, where the lakes are major surface water resources^9^. Changes in lake water quantity will in turn cause fluctuations in their levels^10^. The decline of lake levels directly leads to wetlands losses, vegetation degradation, biodiversity damage, and fishery losses, while extremely high levels can also lead to increased flood risks, soil salinization, and agricultural losses^2,10^. Hence, scientific investigation of lake change is of great significance to understand their water balance, and provide clues on how to regulate these vital water resources.

Many studies about lake level changes had been reported, for example, at Sambhar Lake^11^, Victoria Lake^12^, the Great Lakes^13^, Neusiedl Lake^14^, Qinghai Lake^15^, Poyang Lake^16^, Ebinur Lake^17^, and Nam Co Lake^18^. However, most of these studies focused on lakes in humid regions where lake variations were relatively few. In contrast inland lakes in Central Asia are numerous, however some lakes are drying up, and even some have disappeared. For example, the lake levels of many important water sources have steadily decreased since the 1960s: the Aral Sea has declined by 23 m^1,19,20^, while Manas Lake, and Taitema Lake had disappeared completely since the 1950s and 1970s, respectively^21^. The cause of these lake shrinkages has been attributed to ecological damages and environmental crises^22^.

These endorheic lakes are highly responsive to climate change^23^. Drought indices are an important indicator to reveal the drought and wetness variability of regional climates. Among these, the standardized precipitation index (SPI) and the Palmer drought severity index (PDSI) have been widely used to monitor drought severity^24–26^. The SPI only considers precipitation data without using an evaporative factor, while the PDSI lacks multi-scale characteristics^27–30^. More recently, standardized precipitation evapotranspiration index (SPEI) combines the multi-scalar character of the SPI and the sensitivity of PDSI to changes in evaporative demand^31^. Under the effects of global warming, SPEI has become an effective tool to monitor and study recent climates. For example, Yao *et al*.^32^ suggested an obvious trend towards aggravated drought in Xinjiang based on the SPEI from 1961–2015. Li *et al*.^33^ also found a drying trend over the Central Asia based on the PDSI during the past decade (2000–2014).

In this study, we used the SPEI to analyze an important water source in Central Asia: Bosten Lake, which is the largest inland freshwater lake in China. Recently, the climate and environment of the Bosten Lake Basin experienced significant changes, especially intensified human activities. For example, increasing irrigation and consumption, dam construction, and land desertification caused dramatic changes in the lake’s levels^22,34^. The dramatic change in the lake is not an unusual phenomenon; indeed the same has occurred in the Aral Sea, Balkhash Lake and Ebinur Lake^1,20^. More specifically, the Bosten Lake level has experienced a drastic change, for example, fluctuations as much as 3.7 m occurred during 1988–2003 in this large-scale natural lake^10^. This is different to changes seen in other regions of China and the Tibetan Plateau^35–38^. The cause of these drastic changes is uncertain, but may be attributed, at least in part, to changes in precipitation, evaporation, glacier, river discharge, lake inflow, outflow, and water diversion. However, our knowledge of its water balance and driving components is very limited. In addition, the quantitative fraction of lake water balance change has not yet been assessed. To this end, we conducted a study of the changes of Bosten Lake’s levels, mainly its area and storage, from 1961–2015, to reveal the main driving components in different periods. We present a quantitative analyzes of water balance for this lake.

## Geographical Setting

**Figure 1:**
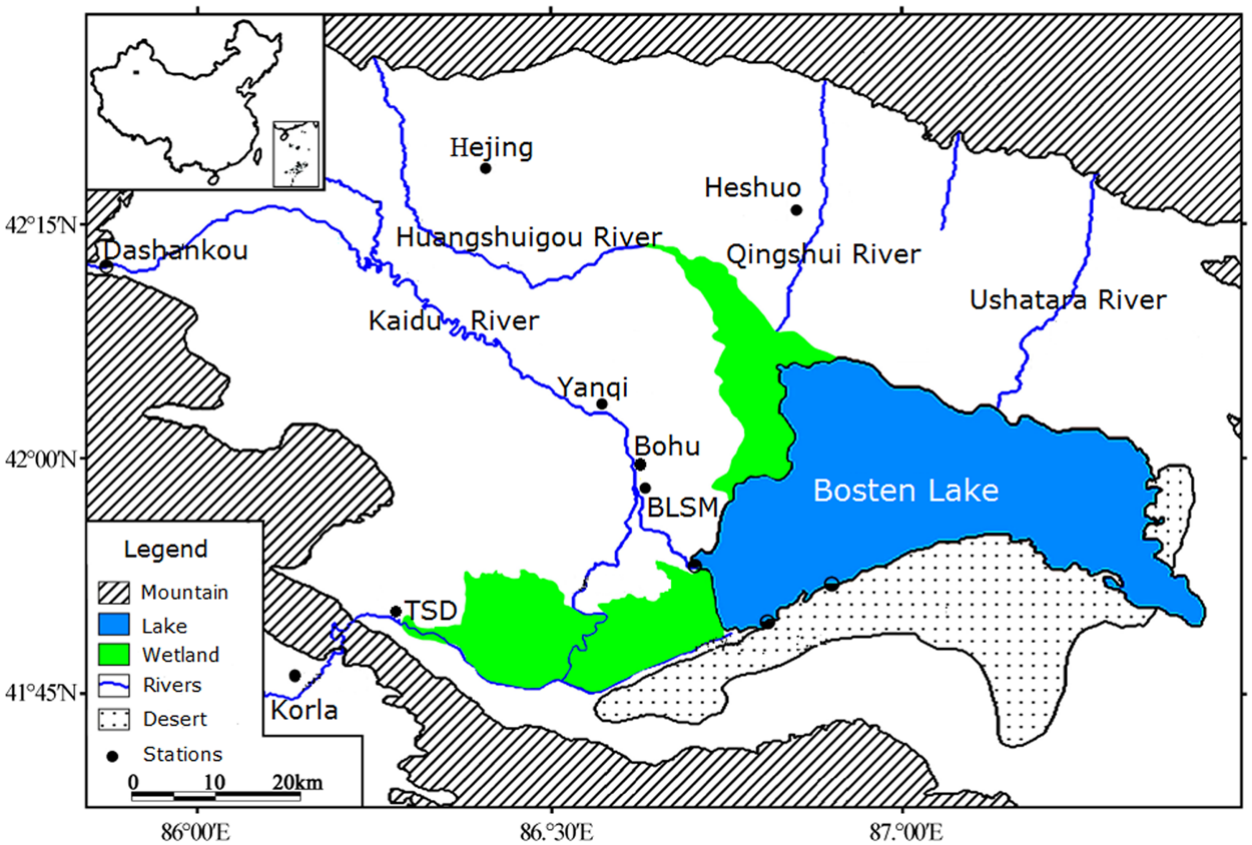
The geophysical location of Bosten Lake, China. TSD is Tashidian hydrological station and BLSM is Baolangsumu hydrological station. The map was created using ESRI ArcGIS 10.2, http://www.esri.com/software/arcgis/arcgis-for-desktop.

Bosten Lake is located in the south of Xinjiang, in an extremely arid region in northwest China (Fig. 1). Bosten Lake is the largest inland freshwater lake in China. The lake area is more than 1,000 km^2^, with a length and width of 55 and 25 km, respectively^39^. The mean lake water depth is 8.2 m with a maximum depth of 17 m^39^. Water feeds into the lake from the Kaidu, Huangshuigou, and Qingshui rivers. Moreover, the Ushatara River flows into the Kongque River, which mainly comes from the Kaidu River, which originates from the Tianshan Mountain region, and contributes about 95% of the total water inflow^40,41^. Lake kept natural outflow states at all times in history until an artificial construction pumping station was built in 1983. Since then, the lake water has been pumped to recharge the Kongque River via a channel^40^. The Bosten Lake basin has an area of 56,000 km^2^, and it belongs to an arid climate zone. The multi-year mean air temperature of the lake area is about 6.3 °C, and the mean annual precipitation is only about 70 mm, while the evaporation is as high as 2000 mm^42^. The lake provides valuable but sparse water resources for irrigation, economic development, and human beings^43,44^. In addition, it also provides an opportunity to divert water to the lower Tarim River^45^.

## Results

### Bosten Lake climatic variability in the past five decades

The PDSI, the SPI and the SPEI are used extensively to assess drought and wetness variability. These indices are formulated by climatic water supply (precipitation) alone or water supply and demand (evaporation)^30,33^. Although drought and wetness variability is primary determined by precipitation, drought severity can be aggravated by higher atmospheric evaporative demand as a consequence of global warming^46^. Calculating the evaporative demand is very critical to monitor drought and wetness variability. The FAO-endorsed Penman–Monteith equation (P–M), is the most popular and physical-based one, employed for the calculation of the potential evaporation (PET)^33^.

**Figure 2:**
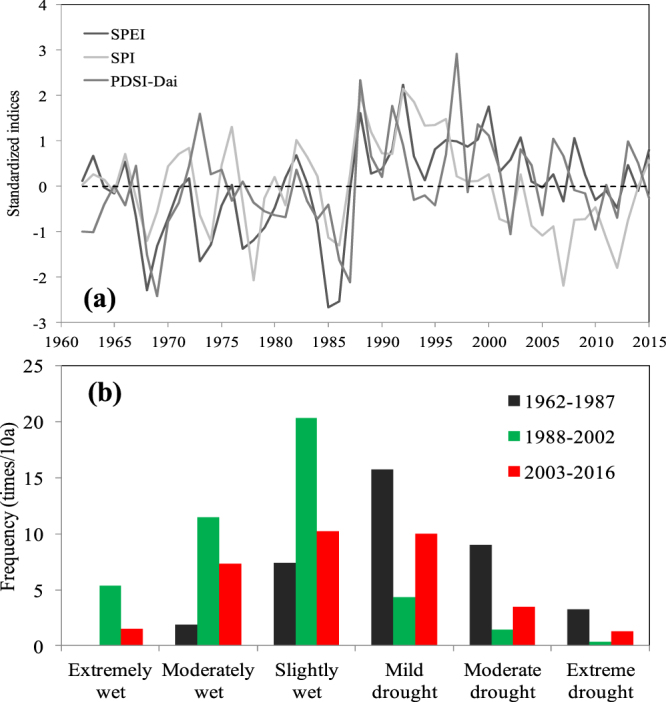
(**a**) Bosten Lake drought and wetness variability based on the SPI, SPEI and sc_PDSI indices between 1961 and 2016 (**b**). Frequency of drought and wetness variability based on the SPEI for the epochs of 1961–1987, 1988–2002 and 2003–2016. The map was created using Matlab 2012a, http://cn.mathworks.com/products/matlab/.

To quantify the recent changes in drought and wetness conditions over the Bosten Lake basin, Fig. 2a shows a time series of the SPEI, SPI and sc_PDSI indices, and Fig. 2b compares the frequency based on the SPEI for the three epochs. As shown in Fig. 2a, the SPEI is a good match with sc_PDSI and SPI. The SPEI had a positive correlation with SPI (R = 0.58, *p* < 0.01) and sc_PDSI (R = 0.47, *p* < 0.01) of the Bosten Lake basin. Furthermore, it illustrates that the three major periods are 1961–1987, 1988–2002 and 2003–2016. The epoch from 1988 to 2002 can be qualified as exhibiting high wetness conditions, while the other two remained at a drought level (Fig. 2a). Despite the smaller overall trend (−0.007 ~ 0.025 per year, *p* > 0.05) in the drought indices from 1961–2016, there was a switch from 1988 onwards to a significantly decreasing trend (−0.023, −0.064 and −0.063 per year at the 0.01 significance level, correspond to SPEI, SPI and sc_PDSI, respectively). SPI-based drought severity has become significantly aggravated relative to that based on the SPEI, which is independent of the effect of the decreased precipitation.

In comparison to the epoch from 1961–1987, intensified wet conditions can be concluded from the wetness category frequency during 1988–2002, where the number of wet months increased from 9.3 to 37.1 months per decade (Fig. 2b). For the period 2003–2016, the drought frequency also increased by a factor of several relative to the period 1988–2002. On average, the drought frequency increased from 6.1 to 14.7 events per decade, and the extreme drought frequency rose rapidly to 1.3 times per decade during 2003–2016 from only 0.3 times per decade during 1988–2002 (Fig. 2b).

### Bosten Lake level, area and storage changes

**Figure 3:**
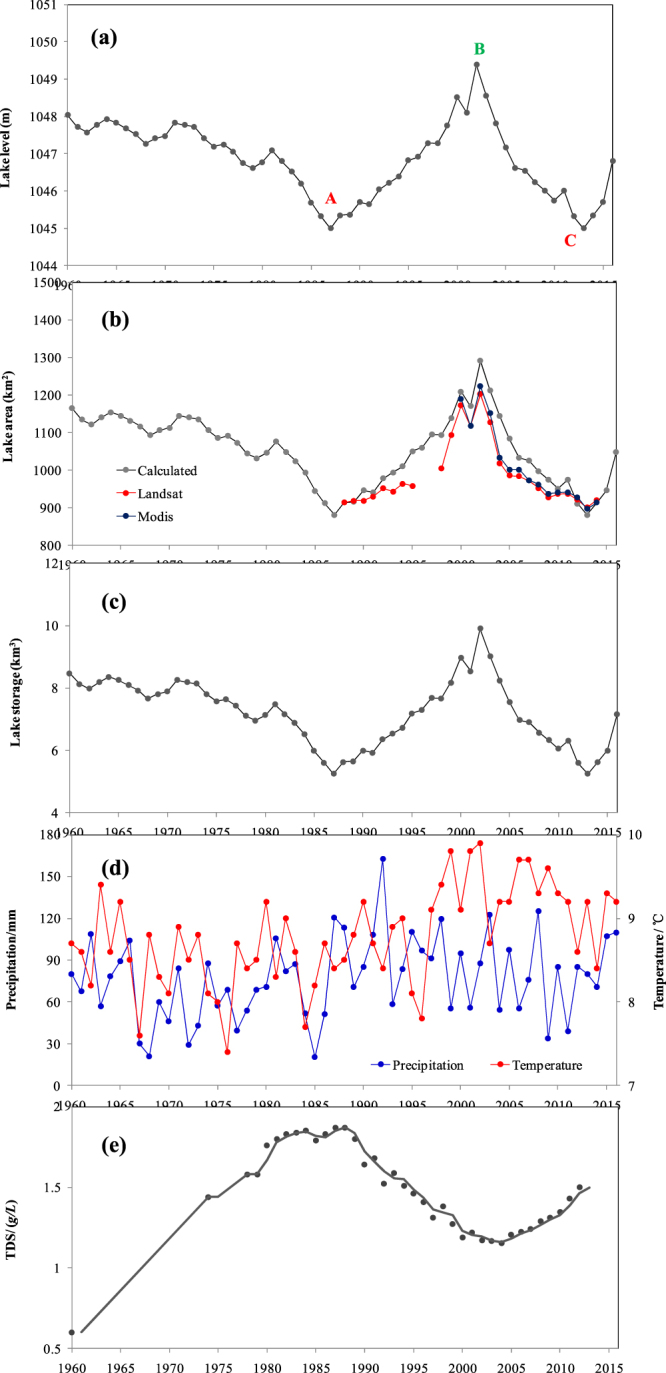
Lake level, area and storage changes in the Bosten Lake between 1961 and 2016; (**a**) A and C represents the minimum level in 1987 and 2013, and B represent the maximum level in 2002 (**b**). Black solid line shows observed lake area, red solid line shows Landsat- derived areas, and blue solid line shows MODIS-derived areas; (**c**) The changes of lake storage between 1960 and 2016. (**d**) Variation of the temperature and precipitation in the lake area from 1960 to 2016. (**e**) Variation of the total dissolved solids (*TDS*) of the Bosten lake from 1960 to 2012. The map was created using Matlab 2012a, http://cn.mathworks.com/products/matlab/.

Figure 3 displays a time series of the mean annual lake level, area, and storage changes in Bosten Lake between 1961 and 2016. The multi-year average of the lake level is 1046.86 ± 1.0 m. The smallest value in the annual mean level was 1045.0 m in 1987 and 2013, while the largest value was 1049.39 m in 2002 (Fig. 3a). In tandem with these changes, the level changes were divided into four epochs: 1961–1987, 1988–2002, 2003–2012, and 2013–2016. Overall, there was a decreasing trend (−0.024 m per year) (Fig. 3a). The lake level presents a decrease between 1961 and 1987, thereafter it shows a dramatic increase, and then a continuous decrease, but a dramatic increase of lake level is mapped in recent years. The average rate of change in the lake levels is −0.083 m per year during 1961–1987, 0.263 m per year during 1988–2002, −0.309 m per year during 2003–2012, and 0.575 m per year during 2013–2016.

The lake area experienced a similar change from 1961 to 2016 (Fig. 3b). The multi-year average of lake area was 1054.46 ± 93.1 km^2^. The rate of change of the surface area in was −7.720 km^2^ per year during 1961–1987, 24.575 km^2^ per year during 1988–2002, −28.898 km^2^ per year during 2003–2012 and 53.779 km^2^ per year during 2013–2016 (Fig. 3b). The lake level changes derived from the Landsat data show a high expansion rate of 19.98 km^2^ per year during 1988–2002, and a shrinkage rate of −14.49 km^2^ per year during 2003–2014 (Fig. 3b). These changes derived from the MODIS data in 2003–2014 show an overall decrease, with a rate of −16.79 km^2^ per year (Fig. 3b).

In addition, the multi-year average of lake storage was 7.22 ± 1.06 km^3^. The rate of change in the water storage in Bosten Lake was −0.088 km^3^ per year during 1961–1987, 0.279 km^3^ per year during 1988–2002, −0.309 km^3^ per year during 2003–2012 and 0.610 km^3^ per year during 2013–2015, respectively (Fig. 3c).

Overall, Bosten lake’s levels, area and storage changes varied in four stages: a decrease (1961–1987), a rapid increase (1988–2002), a drastic decrease (2003–2012) and a recent drastic increase (2013–2016). The drastic changes in the lake levels and area after 1987 accelerated, which could be a driven regime shift arising from regional climate changes^47,48^. Observations from the CMA stations in the lake catchment indicate that temperature and precipitation exhibited a dramatic change during 1961–2015 (Fig. 3d). Temperatures experienced a sharp increase in 1997, and since then remained highly volatile. Precipitation exhibited a sharp increase in 1987, and since then the increasing trend has diminished during the past two decades (Fig. 3d). These changes are consistent with the previous research in the arid regions of China^49–51^. The drought severity was aggravated by a greater evaporative demand caused by the significant temperature rise^32,46^. Dramatic changes in the climatic conditions may bring some adverse effects, and the drought and wetness variability could have played a leading role in altering the lake.

### Quantifying the effects of the hydro-climatic components to runoff changes

Lake inflow is dominated by components that supply the lake with water and regulate changes in its level. Approximately 95% of the total lake inflow to the Bosten Lake comes from the Kaidu River^40,41^. The runoff from Kaidu River is mainly supplied by mountainous precipitation and melting glacier water, which accounts for 61.5% and 38.5%, respectively^51^. Therefore, the runoff change experienced by the Kaidu River is dominated by changes in mountainous precipitation, glacier melting water and evaporation.

The climate elasticity method was used to quantify the effects of the above components to river runoff for the Kaidu River. The values of the climate elasticity coefficient of annual runoff to precipitation $(\frac{\partial R}{\partial P})$, evaporation $(\frac{\partial R}{\partial E{T}_{0}})$ and glacier melting water $(\frac{\partial R}{\partial G})$ are 0.78 ± 0.005, −0.05 ± 0.01 and 0.27 ± 0.01, respectively. This means that a 10% increase in precipitation (glacier melting water) will result in a 7.8% (2.7%) increase in runoff, and that a 10% increase in evaporation will result in a 0.5% decrease in runoff. This illustrates that the change of lake water inflow from the Kaidu River is more sensitive to changes in precipitation and glacier mass loss than to changes of evaporation.

### Bosten Lake Water Balance

**Figure 4:**
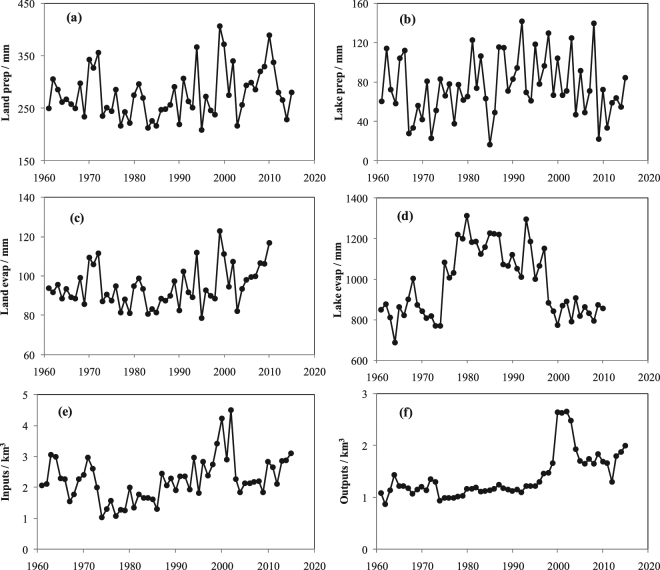
Variation of precipitation, evaporation, lake inputs and outputs during 1961 to 2015 in the Bosten Lake basin. The map was created using Matlab 2012a, http://cn.mathworks.com/products/matlab/.

The Bosten Lake water balance was estimated based on observations of several components, including precipitation (land and lake surface), evaporation (land and lake surface), glacier melting water, and lake input and output. The lake exhibited drastic changes in two phases (1988−2002 and 2003−2015). Variations in the components of the water balance in both phases are listed in Fig. 4 and Table 1. The annual mean and trends of precipitation and evaporation were spatially different in the basin. The average annual precipitation on land surfaces was 287.5 mm and 290.9 mm in both phase (Fig. 4a), while the precipitation on lake surfaces was 91.2 mm and 70.2 mm, respectively (Fig. 4a,b). Precipitation on land surfaces increased significantly in both phases, while the precipitation on lake surfaces decreased. The rate of change in the average annual precipitation on land surfaces was 52.9 mm per year and 13.9 mm per year for the two epochs, respectively, (Fig. 4a), and precipitation on lake surfaces was −11.1 mm per year and −23.5 mm per year, respectively (Fig. 4b). The average annual evaporation on land surfaces was 96.6 mm and 100.3 mm in both phases, respectively, (Fig. 4c), while evaporation from lake surfaces was 1018.2 mm and 841 mm, respectively (Fig. 4d). Evaporation from land surfaces increased during both phases, while evaporation from lake surfaces decreased significantly during 1988–2002, and then weakly increased during 2003–2015 (Fig. 4c,d). The average annual lake input was 2.7 km^3^ and 2.1 km^3^ in both phases, respectively, (Fig. 4e), while the output was 1.5 km^3^ and 1.8 km^3^, respectively (Fig. 4f). The inputs increased significantly at a rate of 0.14 km^3^ per year and 0.05 km^3^ per year in the two phases, respectively, while the outputs increased significantly from 1988–2002, and then weakly decreased during 2003–2015 (Fig. 4e,f). The glaciers’ mass change calculated by the ensemble models decreased at a rate of −0.63 ± 0.31 × 10^3^ kg/m^2^ per year during 1961 to 2015, and −0.68 ± 0.43 × 10^3^ kg/m^2^ per year based on the ICESat data from 2003–2009^52^.

**Table 1:** Lake water balance in Bosten Lake from the 1961 to the 2015.

Water balance components		1961–1987		1988–2002		2003–2015	
		Annual average (km^3^ yr^−1^)	%	Annual average (km^3^ yr^−1^)	%	Annual average (km^3^ yr^-1^)	%
*ΔL*		−0.12		0.31		−0.48	
*Lake water supply*	***P***_***L***_	0.29	6	0.31	5	0.31	6
	***R***_***in***_	1.41	31	1.98	35	1.59	29
	***G***	0.52	12	0.73	13	0.59	11
*Loss of lake water*	***E***_***L***_	1.05	−23	1.06	18	0.88	−16
	***R***_***out***_	1.15	−25	1.55	27	1.83	−33
***R***_***g***_ **±** ***ε***		−0.13	−3	−0.1	2	−0.26	−5

The lake water balance components include changes in the lake water storage (*Δ****L***), lake precipitation (***P***_***L***_), runoff generated by precipitation (***R***_***in***_), melting glacier water (***G***), lake evaporation (***E***_***L***_), lake outflow (***R***_***out***_), and groundwater exchange and errors (***R***_***g***_** ± *ε***).

**Figure 5:**
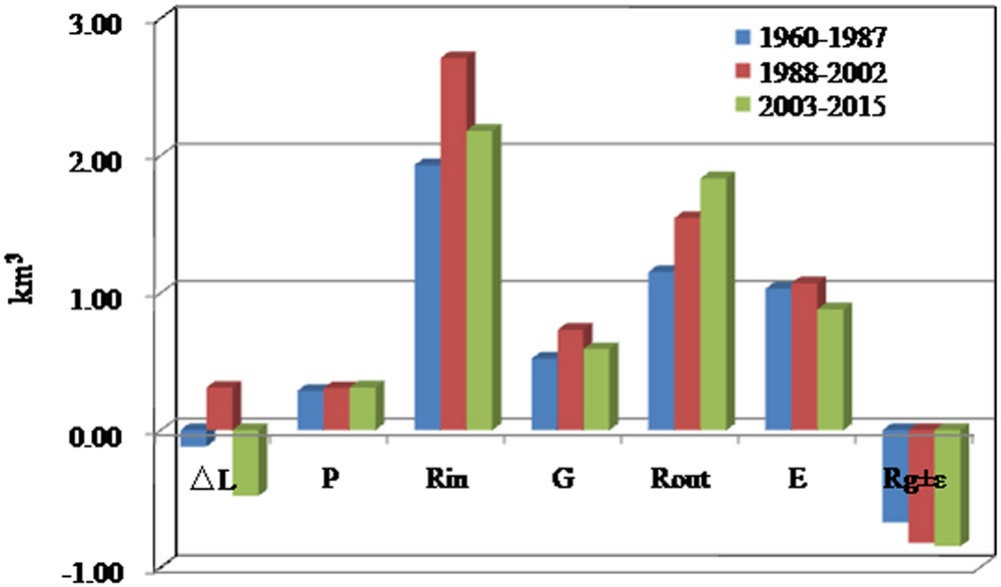
Water balance in three phases over the Bosten Lake. The lake water balance components include lake precipitation (***P***_***L***_), runoff generated by precipitation (***R***_***in***_), glacier melting water (***G***), lake evaporation (***E***_***L***_), lake Outflow (***R***_***out***_), groundwater exchange and errors (***R***_***g***_ ± ***ε***) and average change in lake storage (ΔL). The map was created using Matlab 2012a, http://cn.mathworks.com/products/matlab/.

Lake water balance components for the Bosten Lake in three phases (1960–1987, 1988–2002 and 2003–2015) were listed in Fig. 5. The mean annual precipitation over the lake was 0.29, 0.29 and 0.31 km^3^ in three phases (similarly hereinafter), respectively, surface runoff water input generated by precipitation was 1.93, 2.71 and 2.18 km^3^, glacier melting water into the lake was 0.52, 0.73 and 0.59 km^3^, evaporation from the lake was 1.03, 1.07 and 0.88 km^3^, water outflow from the lake was 1.15, 1.55 and 1.83 km^3^, groundwater inflow and error was −0.67, −0.82 and −0.85 km^3^, and the change in lake storage was −0.12, 0.31 and −0.48 km^3^. This means that lake water increased due to the increased water input (R_in_ and G) higher than water output (E_L_ and R_out_) for the 1988–2002. In 2003–2015, the lake water decreased was caused by the decreased runoff (R_in_ and G) and increased water output (R_out_).

## Discussion

### Climate-driven regime shift and their influence to lake changes

**Figure 6:**
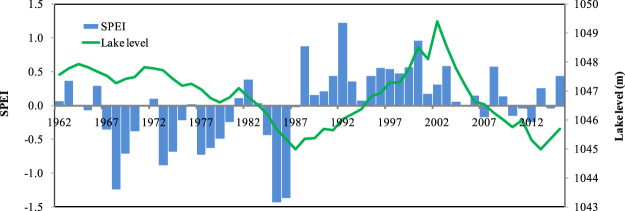
Lake level and SPEI variability in the Bosten Lake between 1961 and 2016. The map was created using Matlab 2012a, http://cn.mathworks.com/products/matlab/.

Over the past 50 years, studies showed that the climate exhibited a dramatic change in northwestern China, which is becoming warmer and wetter^47,48,53–55^. Dramatic changes in the climatic conditions may bring some adverse effects^32^, where the dramatic fluctuations of Bosten Lake’s levels are a case in point. In this study, we compared the time series and frequency between the SPEI, SPI and sc_PDSI indices, and found that SPEI is a good match with sc_PDSI and SPI. Actually, SPEI is an effective tool to monitor recent climates under the global warming, due to it combines the multi-scalar character of the SPI and the sensitivity of PDSI to changes in evaporative demand^31^. The lake level change is highly similar to the SPEI time series, lake level rose rapidly from 1988, and the SPEI increased at the same time (Fig. 6). SPEI exhibited a sharp increase in 1988, and since then the increasing trend decreased during the past two decades. Meanwhile, the drought severity was aggravated by a significant rise in temperature coupled with an insignificant increase in precipitation^32^. Undoubtedly, while climate-driven regime shifts contribute a part of the lake level changes, and there are also other factors including ecological water conveyance, agricultural irrigation and water consumption.

### Retreating glaciers and dramatic lake changes

The river runoff from the Tianshan Mountains heavily depends on glaciers (snow), hence glacier meltwater occupies an important role in the total amount of river discharge^50^. Glacier melt and their retreat in northwest China has been accelerated by global warming, where 82.2% of glaciers are retreating, and their total area has decreased by 4.5%^56^. The retreating trends have been exacerbated since the 1990s^57^, and the Bosten Lake Basin is no exception.

**Figure 7:**
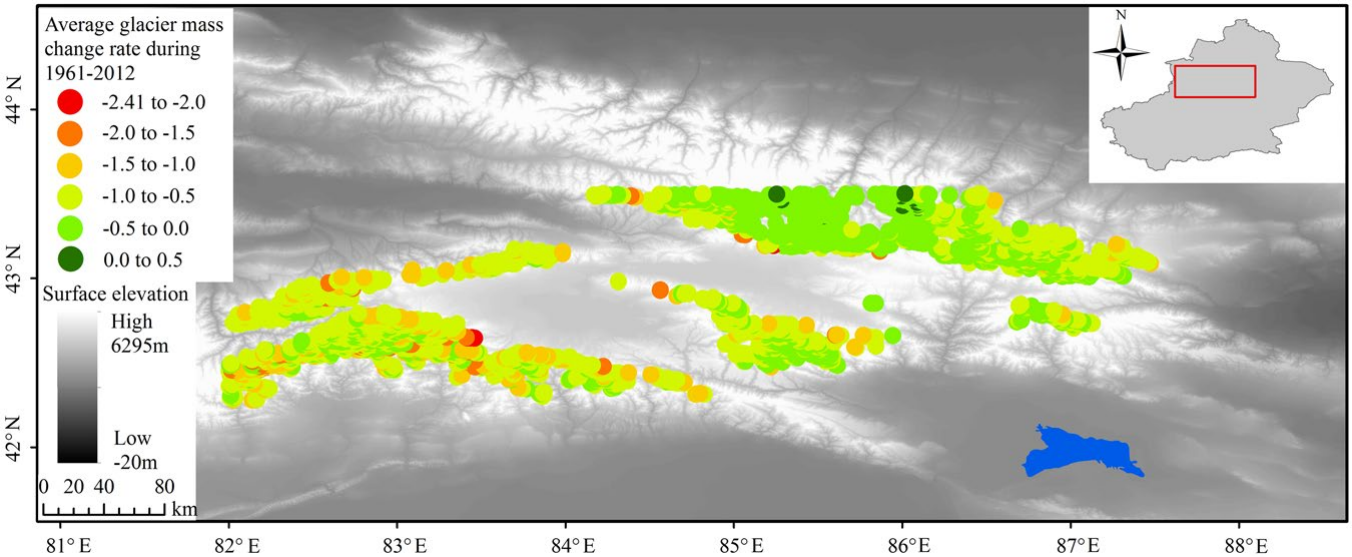
Glacier mass change rates in the Bosten Lake basin. Each coloured dot represents one glacier, the colour depicting the average mass change rate for the period 1961–2012 as calculated with the ensemble of models reported by the Farinotti *et al*.^52^. The map was created using ESRI ArcGIS 10.2, http://www.esri.com/software/arcgis/arcgis-for-desktop.

From the climate elasticity method and lake water balance estimate in Bosten Lake, glaciers are a key factor for lake expansion/retreat. Indeed, glaciers show a retreating trend in the Bosten Lake Basin during 1963 to 2000. The decrease in glacier area is 38.5 km^2^, and the decrease rate was 0.31%/a^56^. Table 2 provides an overview of the glacier area and volume changes in the Bosten Lake Basin. The glacier area and volume changes rate are −15.3% and −19.5% during the 1960s to 2000s, respectively. Most of the glaciers in the basin are small-scale ones, which account for 72% of the total number of glaciers^58^. There is an obvious retreat for small-sized glaciers (glacier area <1 km^2^), where the glacier area and volume changes rate were 23.9% and 31.4% during 1963 to 2004, respectively^58^. Figure 7 shows the average glacier mass change rates in the Bosten Lake Basin during 1961 to 2012 as calculated with the ensemble of models. The average glacier mass change rate was −0.63 ± 0.31 × 10^3^ kg m^−2^ yr^−1^ during 1961 to 2012. In the period 2003−2009, the average glacier mass change rates was −0.69 ± 0.28 × 10^3^ kg m^−2^ yr^−1^ and −0.68 ± 0.43 × 10^3^ kg m^−2^ yr^−1^ as calculated from the models and the ICESat data, respectively^52^. From the analyses of glacier changes, the lake area dramatically expanded from 1988 to 2002, and dramatically shrank from 2003 to 2015, while showing an overall decrease in the total glacier area and mass from 1961 to 2012. Therefore, the dramatic changes of the lake, and the continuous glacier retreat are not coupled in the Bosten Lake Basin. It was also suggested that the changes experienced by Bosten Lake were dramatic and caused primarily by variations in the precipitation in the basin, and secondarily by the accelerated rate of melting glaciers. Similar results were found in the Yamzhog Yumco Basin, Tibetan Plateau^59^.

**Table 2:** Overview of the data used for quantifying glacier area and volume changes in the Bosten Lake Basin.

Name	Study period	Glacier number	Glacier area change		Glacier volume change		Source
			km^2^	/%	km^3^	/%	
Kaidu River	1963–2000	853	−38.5	−15.9	—	—	Liu *et al*.^56^
Albin mountains	1963–2000	70	−6.84	−12.5	—	—	Li *et al*.^76^
5Y692	1963–2004	92	−14.17	−17.4	−0.99	−19.5	Gao *et al*.^58^

Glacier recession rates were obviously larger in the 2000s than in previous epochs, which results from the continuously rising temperatures. From 1961 to 2016, the annual mean temperature, maximum temperature and minimum temperature all experienced a significant increasing trend in the Tianshan Mountains, with rates of 0.34 °C/10a, 0.19 °C/10a and 0.56 °C/10a, respectively. Meanwhile, substantial glacier mass loss in the Tianshan Mountains was reported over the past 50 years^52^. A study suggested that the temperature increase had a large effect on the melting of small scale glaciers^60^. Thus, the rising temperatures clearly accelerated the rate of melting glaciers due to the dominance of small-sized ones.

### Lake changes and water balance estimates

According to the above analyses, precipitation and glacier melting water are the main sources of recharge for lake water input, and influence whether a lake expands or retreats.

Table 1 lists the average values of the Bosten Lake water balance components and their proportions between 1961 and 2015. Lake precipitation, inputs generated by precipitation, and glacier melting water accounted for 10.27%, 65.56% and 24.17% of the total lake water supply during 1988−2002, and 12.45%, 63.86% and 23.69% during 2003−2010, respectively. Therefore, precipitation is the dominant factor for supplying the lake, which reached 75.83% and 76.31% during 1988−2002 and 2003−2015, respectively. However, our analysis also shows that the precipitation increased by 0.48%, while the glacier melting water decreased between the two phases. Evaporation and output accounted for 35.43% and 51.32% of the lake water loss during 1988−2002, and 35.34% and 73.49% during 2003−2015, respectively. Therefore, the slight change in evaporation during both phases was not able to reduce the lake water as the increased input of lake water was much more than the increased loss arising from evaporation.

The average lake water supply increased by 0.8 km^3^ per year during 1988 to 2002 in comparison with that in 1961−1987. The portion derived from precipitation (including lake surfaces and generated runoff) and glacier melting water contributed 73.75% and 26.25% of the total supply. Indeed 5% and 50% of water was lost due to an increase in evaporation and output, while 53.75% of them contributed to the lake enlargement. The average lake water storages decreased by 0.79 km^3^ per year during 2003 to 2015 in comparison with that in 1988–2002. Among the decreased supply, the amount of precipitation and glacier melting water was 0.39 km^3^ per year and 0.14 km^3^ per year, respectively. Meanwhile, the lake water output had increased by 0.28 km^3^ per year, while the evaporation decreased by 0.19 km^3^ per year.

### Irrigation and water conveyance exacerbated lake water shortage and degradation

**Figure 8:**
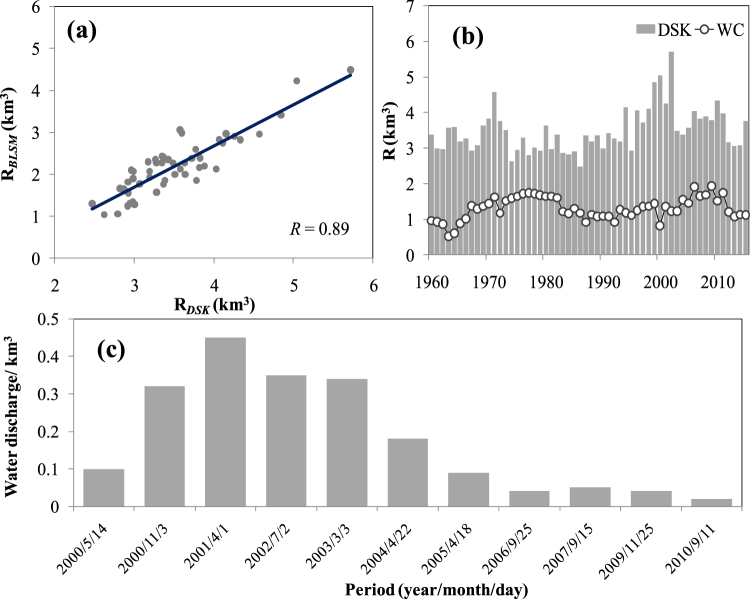
(**a**) The relationship between annual runoff (*DSK*) and lake inflow (*BLSM*) from 1961 to 2015. (**b**) Variation of annual runoff (*DSK*) and water consumption (*W*_*C*_) in Yanqi Oasis during 1961 to 2015. (**c**) Water diversion from Bosten lake to the lower reaches of the Tarim River from 2000 to 2015. The map was created using Matlab 2012a, http://cn.mathworks.com/products/matlab/.

The annual runoff from the Kaidu River contributes about 95% of the total lake water input^40,41^. Figure 8a also shows has a clear positive correlation between the river runoff and lake inflow. However, water consumption in the Yanqi Oasis clearly influences the lake water inputs via agricultural irrigation, industrial and domestic water use. Figure 8(b) displays the variations of annual water consumption and river runoff during 1961 to 2010. The average annual river runoff totaled 3.51 km^3^, however, the average annual water consumption in the basin was 1.31 km^3^, and water consumption accounted for 37.3% of the total river runoff. For the different periods, water consumption accounted for 40.4%, 30.2% and 42.5% of the total river runoff for the three periods mentioned above, respectively. The Bosten Lake Basin is an irrigation agricultural area, and is mostly composed of farmland. The area of the farmland was about 5.47 × 10^4^ ha in 1990, and then it grew rapidly in recent years, reaching a value of 11.75 × 10^4^ ha in 2010^51^. Hence irrigation accounted for 90% of the total water consumption in the lake basin^61^. All these indicate that water consumption accounts for over one half of the total river runoff, implying that water consumption by irrigation has largely influenced the dramatic changes seen in the lake basin.

Bosten lake basin is a main source of the Tarim River, which is the longest inland river in China, and has undergone major ecological degeneration caused by unbridled utilization of water resources^62,63^. The Chinese government implemented the Ecological Water Conveyance Project (EWCP) in 2000 in order to recover the “Green Corridor” in the lower reaches of the Tarim River^64,65^. The EWCP transferred water from the Bosten Lake to the Daxihaizi Reservoir, and finally to the Taitema Lake^64^. The EWCP implementation significantly increased groundwater levels and effectively restored degraded vegetation^66,67^. However, water used by the EWCP was mainly provided by the Bosten Lake and irrigation saving water, which further exacerbated water scarcity in the Bosten Lake Basin. The total ecological water taken from Bosten Lake was 1.91 km^3^ during 2002 to 2010, and stop watering after 2010 due to low-flow years (Fig. 7c). The total dissolved solids (*TDS*), synonymous with the water salinity, is a key indicator of the water environment^68^. Its changes in Bosten Lake also experienced three stages: a rapid increase (1960–1987), a drastic decrease (1988–2002), and a recent increase (2003−2015), but it showed a clearly anti-phase with the lake level changes (Fig. 3e). This shows that the TDS decreased as lake levels increased. The smallest value of TDS was 0.6 g/L in 1960 while the largest value was 1.87 g/L in 1987 (Fig. 3e). The lake has experienced salinization due to the large-scale water reclamation in the source areas, excessive exploitation and utilization of water resources, reduction of inflow and increase of salt flux into the lake^44^. The TDS changes are significantly (negatively) correlated with changes in lake inflow, lake outflow and lake level, with correlation coefficients of −0.51, −0.84 and −0.69 (*p* < 0.01), respectively. The TDS changes were dominated by the lake level, and the wastewater emissions is also an important factor affecting salinization of the lake. A correlation analysis showed significant (positive) correlations between the TDS changes and total wastewater emissions and industrial wastewater, with correlation coefficients of 0.90 and 0.86 (*p* < 0.01), respectively^42^. Wastewater emissions were about 3.45 × 10^4^ t in 2001, which then grew rapidly in recent years, reaching a value of 7.32 × 10^4^ t in 2010^42^. The industrial wastewater emissions exceeded more than 50% of the total wastewater emission in the lake basin^42^. The wastewater emissions lead to the increase of salt flux into the lake, expediting its salinization, and finally restricting the freshwater environment.

All these demonstrate that irrigation and water conveyance exacerbated the water shortages in the Bosten Lake Basin, and that wastewater emissions gave rise to water salinization. The intensity of human disturbances in the Bosten Lake Basin was 62~67.7% before 2000, and reaching more than 80.8% in the 21 century^42^. The water system is clearly fragile in the Bosten Lake Basin, and the uncertainty of volatile water resources is exacerbated by global warming. However, human activity is largely changing the natural water cycle system in the Bosten Lake basin. The future of Bosten Lake is largely dependent on human activities.

## Conclusions

Bosten Lake, the largest inland freshwater lake in China, has experienced drastic change over the past five decades. This study aimed to identify annual changes in the lake’s water components and investigate water balance of the lake from 1961 to 2016. The multi-year average of the lake level is 1046.86 ± 1.0 m. The lake level presents a decrease between 1961 and 1987 (−0.083 m), thereafter it shows a dramatic increase (0.263 m/a during 1988–2002), and then a continuous decrease (−0.309 m/a during 2003–2012), but a dramatic increase of lake level is mapped in recent years (0.575 m/a during 2013–2016). The lake area and storage experienced a similar change from 1961 to 2016. Lake water balance and climate elasticity method estimated that the lake precipitation, inputs generated by precipitation, and glacier melting water accounted for 10.27%, 65.56% and 24.17% of the total lake water supply during 1988−2002, and 12.45%, 63.86% and 23.69% during 2003−2010, respectively. Evaporation and output accounted for 35.43% and 51.32% of the lake water loss during 1988−2002, and 35.34% and 73.49% during 2003−2015, respectively. Climate-driven regime shifts contribute a part of the lake level changes, and there are also other factors including ecological water conveyance, agricultural irrigation and water consumption. During 2003 to 2012, implementation of the ecological water conveyance project (EWCP) significantly increased the lake’s outflow, while a decreased precipitation led to an increased drought frequency. Furthermore, wastewater emissions may give rise to water degradation, human activity is completely changing the natural water cycle system in the Bosten Lake.

## Methods

### Lake Water Balance

The Bosten Lake is an open lake with several outlets; therefore any changes in the lake’s overall water level (*ΔL*) includes precipitation over the lake’s surface (*P*_*L*_), surface runoff inflow to the lake (*R*_*in*_), glacier melting water (*G*), evaporation from the lake surface (*E*_*L*_), water outflow from the lake (*R*_*out*_), and the groundwater inflow and its error (*R*_*g*_ ± *ε*). The lake water balance model can be expressed as:

1 $$\Delta L=P+{R}_{in}+G-{E}_{L}-{R}_{out}+{R}_{g}\pm \varepsilon $$

### Climate Elasticity Method

For a given catchment, the change of river discharge (*R*) for different phases can be expressed as:

2 $${\rm{\Delta }}R={\rm{\Delta }}{R}_{C}+{\rm{\Delta }}{R}_{H}$$

where Δ*R*_*C*_ and Δ*R*_*H*_ are changes in *R* arising from climatic variability and human disturbances, respectively.

The Kaidu River basin is in an uninhabited natural system because human disturbance is very slight^42,69^. Runoff is mainly supplied by mountainous precipitation and melting glacier water^42^. Therefore, the change in *R* is dominated by climatic variability (Δ*R*_*C*_), which can be approximately estimated as:

3 $${\rm{\Delta }}{R}_{c{\rm{limate}}}=\frac{\partial R}{\partial P}{\rm{\Delta }}P+\frac{\partial R}{\partial E{T}_{0}}{\rm{\Delta }}E{T}_{0}+\frac{\partial R}{\partial G}{\rm{\Delta }}G$$

where Δ*P*, Δ*ET*_0_ and Δ*G* are changes in the amount of precipitation, evaporation and glacier melting water, respectively. The climate elasticity coefficients, $\frac{\partial R}{\partial P}$, $\frac{\partial R}{\partial E{T}_{0}}$, and $\frac{\partial R}{\partial G}$ are the contributions of changes in *P*, *ET*_0_ and *G* to *R*, respectively. Therefore, these relationships can be expressed as:

4 $$\frac{\partial R}{\partial P}+\frac{\partial R}{\partial E{T}_{0}}+\frac{\partial R}{\partial G}=1$$

Based on the Budyko hypothesis^70^, the climate elasticity coefficients can be calculated as follows:

5 $$\frac{\partial R}{\partial P}={({P}^{\bar{w}}+E{{T}_{0}}^{\bar{w}})}^{(\frac{1}{\bar{w}}-1)}{P}^{\bar{w}-1}$$

6 $$\frac{\partial R}{\partial E{T}_{0}}={({P}^{\bar{w}}+E{{T}_{0}}^{\bar{w}})}^{(\frac{1}{\bar{w}}-1)}E{{T}_{0}}^{\bar{w}-1}-1$$

Thus, the contributions of changed *G* to changed R can be expressed as:

7 $$\frac{\partial R}{\partial G}=1-\frac{\partial R}{\partial P}-\frac{\partial R}{\partial E{T}_{0}}$$

### Trend analysis

We used the nonparametric Mann–Kendall method (M–K) recommended by the WMO^71–73^ to investigate the trends of the hydro-meteorological factors in Bosten Lake from 1960 to 2016. The Pearson correlation coefficient was used to establish the statistical significance of relationships between any two factors.

### Data analysis

The Bosten lake level data from 1960–2016 were collected from the Xinjiang Environmental Protection Academy of Science in China (http://www.xjaeps.com/). The annual lake level averaged from monthly data. The lake area (storage) data from 1960–2016 were estimated by the water level-water area (water level–water storage) curve based on on-spot observations obtained in 2000^21^. The Landsat- and MODIS-derived areas data from 1988–2014 and 2000–2014 were obtained from the work of Sun *et al*.^74^. The quality of the Landsat- and MODIS- data was assessed against available observations^74^. The total dissolved solids (*TDS*) of Bosten Lake from 1960–2012 were collected from the Xinjiang Environmental Protection Academy of Science in China (http://www.xjaeps.com/).

The monthly meteorological data were providing by the China Meteorological Data Service Center (http://data.cma.cn/) for five ground-based meteorological stations (Korla, Yanqi, Hejing, Heshuo and Bohu station) for the periods of 1961–2015. Mean data from the 5 stations were used for the basin-scale analysis. The original monthly data includes the air temperature, precipitation, maximum temperature, minimum temperature, pressure, and relative humidity. To guarantee consistency, the monthly data were checked to ensure that they met the expected standards. The standard requires strict quality control processes including extreme inspection, time consistency check, and others before releasing these data. The annual observed data of the river’s discharge, lake inflow and outflow series (1961–2015) were collected from the Dashankou (*DSK*), Baolangsumu (*BLSM*) and Tashidian (*TSD*) controlling sites from the Xinjiang Tarim River Basin Management Bureau in China (http://www.tahe.gov.cn/Category_1/Index.aspx). The pan evaporation measurements were taken from the Yanqi meteorological station collected from Xinjiang Meteorological Bureau, whose measurements ended in 2009. These data were used to represent the evaporative demands of the atmosphere instead of actual evaporation from the lake’s surface. Traditionally, the actual amount of evaporation was determined using the pan evaporation multiplied by a conversion coefficient, which was set to 0.47 for Bosten Lake^21^.

In this paper, we used the PDSI^24,75^, the SPI^25^ and the SPEI^26^ to assess the drought and wetness variability. The potential evaporation (PET) is a key factor when calculating the PDSI and SPEI values. To calculate the PET based on the Penman–Monteith model, we collected measurements of the air temperature, maximum temperature, minimum temperature, pressure, relative humidity from the meteorological stations in the lake basin. The meteorological data in this study are providing by the China Meteorological Data Service Center (http://data.cma.cn/). The self-calibrating PDSI (sc_PDSI) was reported by the Dai *et al*.^75^, with the spatial resolution of 0.5 degree (https://www.esrl.noaa.gov/psd/data/gridded/data.pdsi.html). We used the regional average sc_PDSI value in the lake basin region (40.5°N-42°N, 85°E-88°E). More detailed information on the calculation of the three drought indices are presented by Vicente-Serrano *et al*.^46^, Dai *et al*.^75^ and McKee *et al*.^25^.
